# Management of a spontaneous supra-aortic arterial dissection: a case report

**DOI:** 10.1186/s13256-021-02886-3

**Published:** 2021-06-02

**Authors:** Omar M. Sharaf, Tomas D. Martin, Eric I. Jeng

**Affiliations:** 1 College of Medicine, University of Florida, 1600 SW Archer Rd m509, Gainesville, FL 32610 USA; 2 Division of Cardiovascular Surgery, College of Medicine, University of Florida, 1600 SW Archer Rd, Gainesville, FL 32607 USA

**Keywords:** Innominate artery dissection, Malignant hypertension, Spontaneous dissection

## Abstract

**Background:**

Acute DeBakey type I and type II aortic dissections are indications for emergent surgical repair; however, there are currently no standard protocols in the management of isolated supra-aortic dissections. Prompt diagnosis and management of an isolated innominate artery dissection are necessary to prevent distal malperfusion and thromboembolic sequelae.

**Case presentation:**

A 50-year-old Caucasian gentleman presented with chest pain radiating to his jaw and right arm. He had no recent history of trauma. On physical exam, he was neurologically intact and malignantly hypertensive. Computed tomographic angiography of the chest and neck confirmed a spontaneous isolated innominate artery dissection without ascending aorta involvement. Given the lack of evidence for rupture, distal emboli, and/or end-organ malperfusion, the decision was made for initial non-operative management—anti-impulse regimen, antiplatelet therapy, and close follow-up.

**Conclusions:**

Medical management of a spontaneous isolated innominate artery dissection is appropriate for short-term and potentially long-term therapy. This not only spares the patient from a potentially unnecessary surgical operation but also provides the surgeon and the patient the time to plan for a surgical approach if it becomes necessary.

## Background

Aortic dissections are caused by multiple etiologies including connective tissue disorders, illicit drug use, trauma, and/or uncontrolled hypertension. Acute DeBakey type I and II aortic dissections are classically managed with excision of the intimal tear with reconstruction of the aorta and postoperative long-term blood pressure control with anti-impulse therapy. Uncomplicated DeBakey type III aortic dissections have historically been managed non-operatively with strict blood pressure control regimens. On this spectrum are isolated uncomplicated supra-aortic dissections. They can be classified in two major groups: traumatic—due to an inciting event such as blunt chest trauma, deceleration events, and sports injuries; and/or spontaneous—when no inciting cause is identified [1, 2]. Supra-aortic dissections can involve either single or multiple arch vessels, and the mean age for its occurrence is 45 years [2]. As a result of the rare nature of this condition, there are no uniformly approved guidelines for managing uncomplicated spontaneous isolated innominate artery dissection [2].

## Case presentation

**Fig. 1:**
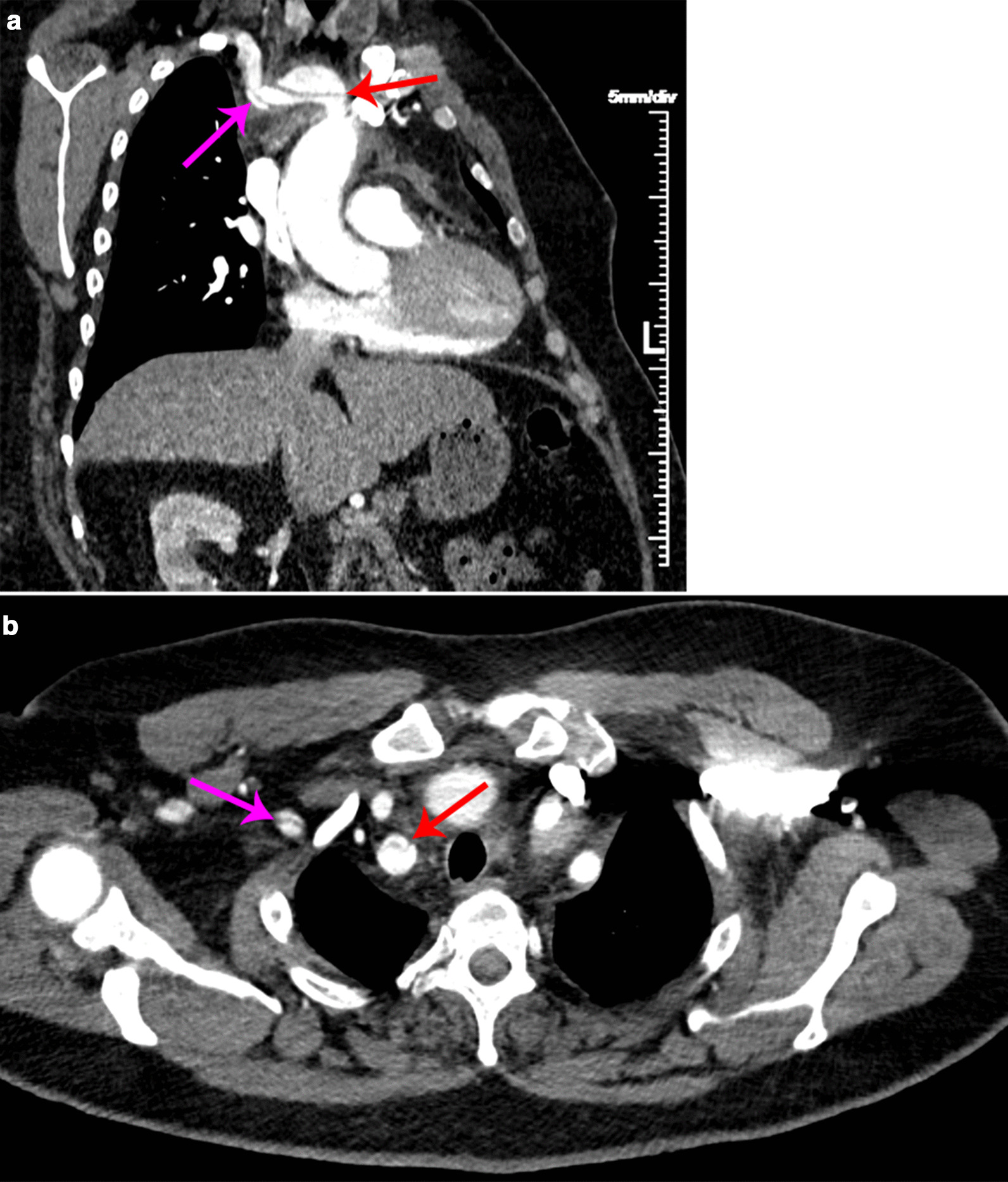
Computed tomographic angiography chest results at initial admission. Innominate artery demonstrates distinct widening on coronal oblique view (**a**) after takeoff from the aortic arch. This (red arrow, **a**) represents the origin of dissection, which separates the true lumen (below red arrow, **a**) from the false lumen (above red arrow, **a**). Extension distally into the subclavian artery is observed (pink arrow, **a**). Axial imaging (**b**) shows true and false lumens in both the subclavian artery (red arrow, **b**) and the axillary artery (pink arrow, **b**), evidencing distal extension of dissection. Aortic involvement is absent—no true and false lumens are identified.

**Fig. 2:**
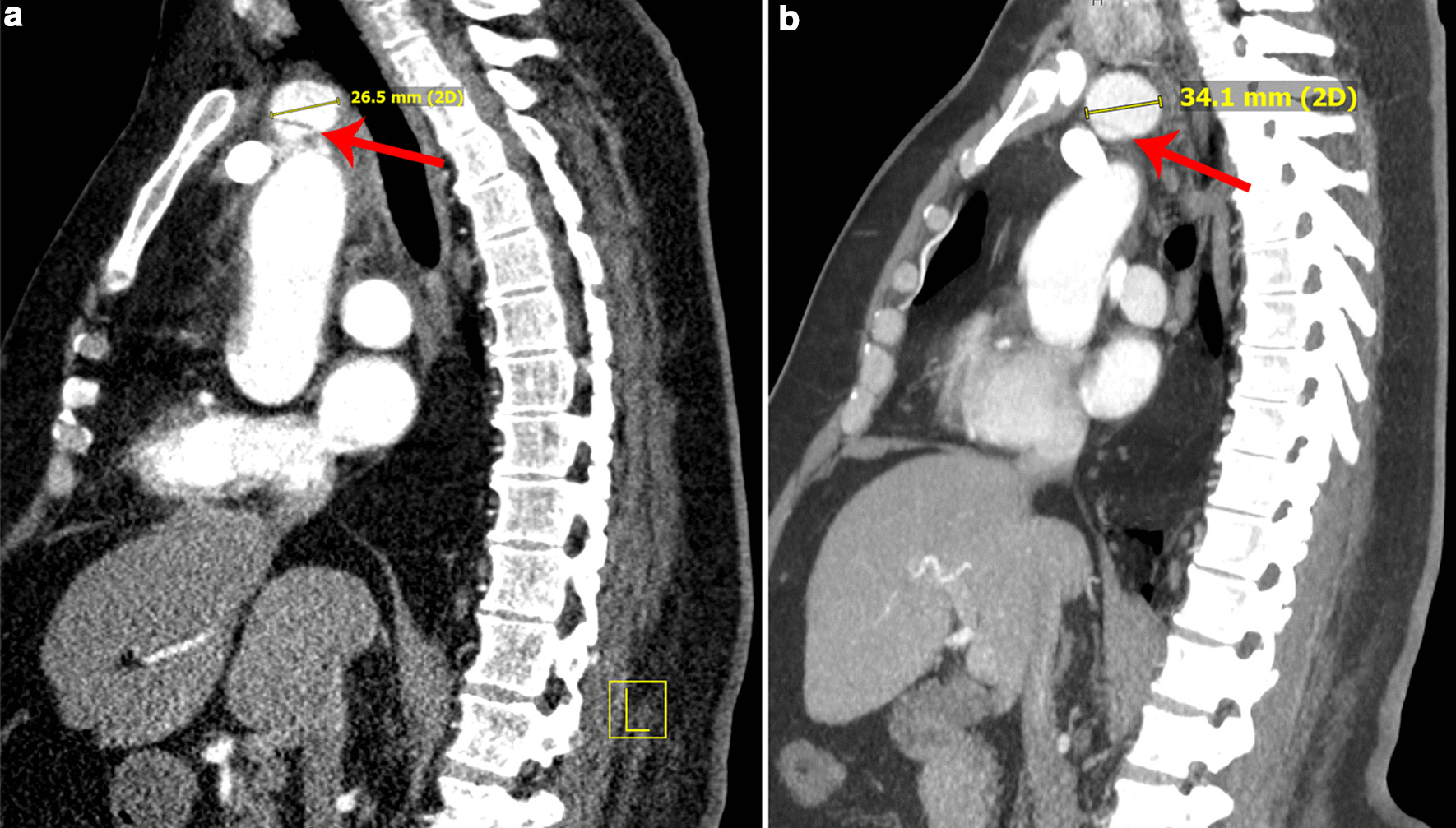
Sagittal computed tomographic angiography of the chest and neck at initial presentation (**a**) and at 3-month follow-up (**b**). Imaging reveals slightly increased diameter of the innominate artery after takeoff from the aortic arch compared to diameter at initial presentation. True and false lumens are separated by intimal flap (red arrows, **a** and **b**).

A 50-year-old morbidly obese (body mass index 44.16 kg/m^2^) Caucasian gentleman with uncontrolled hypertension presented to a nearby emergency department with shortness of breath and sudden-onset, intermittent, substernal chest pain radiating to both sides of his jaw and to his right arm. He had an intact neurological examination and was without focal or motor deficits. Past medical history is negative for prior cardiovascular interventions, and his family medical history was negative for cardiovascular and/or aneurysmal disease. He denies cocaine and/or illicit drug use, and was a 20-pack-year smoker who quit 7 years prior. On examination, the patient was malignantly hypertensive at 261/142 mmHg and with sinus tachycardia at a heart rate of 121 beats per minute. His work-up consisted of an electrocardiogram which showed sinus tachycardia with no ST changes, a troponin level of 0.019 ng/mL within normal limits, and computed tomographic angiography (CTA) revealing an innominate artery dissection with extension distally through the right subclavian artery into the opening of the axillary artery (Fig. 1). He was transferred to the University of Florida Health—Aortic Disease Center where the patient was treated with continuous intravenous infusions of clevidipine and esmolol for anti-impulse therapy (titrated to goal systolic blood pressure of less than 120 mmHg, heart rate less than 80 beats per minute) to prevent worsening dissection, and aspirin [3] to minimize risk of thromboembolic sequelae. At 24 hours from presentation a repeat CTA of the chest and neck was performed, confirming an unchanged dissection of the innominate artery without propagation into the ascending aorta or aortic arch. Transthoracic echocardiography showed normal right and left ventricular systolic and diastolic function with no hemodynamically significant valvular dysfunction. The patient was subsequently discharged 72 hours after admission without pain or symptoms, on an oral medication regimen of amlodipine (5 mg daily), labetalol (600 mg three times a day), and aspirin (81 mg daily). He presented to his 3-month, 6-month, and 1-year follow-up clinic appointments with adequate blood pressure control equal bilaterally at 122/80 mmHg, no evidence of upper extremity muscle atrophy, and no neurologic deficits. Interval CTA showed aneurysmal enlargement of the innominate artery from 2.65 to 3.41 cm (Fig. 2). The decision was made to continue interval medical management with amlodipine, labetalol, and aspirin, with close follow-up and a plan for surgical intervention if aneurysmal growth persists and/or debilitating symptoms present.

## Discussion and conclusions

Surgical treatment is the standard of care for arterial dissection involving the ascending aorta [4] to protect against proximal ascending aorta sequelae, including acute aortic insufficiency, coronary malperfusion, rupture, and tamponade. Reported cases of supra-aortic dissection varied in presentation and thus management. In one of the largest series reported of 27 patients over three decades, Kieffer* et al*. [5] found excellent results with surgical management. While some reported their surgical interventions when there was concern for rupture [6], others argue for early surgical intervention in this entity [7]. Some groups recommend medical management when vessel diameter is less than 3 cm and there is no evidence of distal emboli or malperfusion [8, 9]. Unless there is evidence of persistent growth, rupture, and/or neurologic deficit related to dissection flaps, we treat acute presentations of supra-aortic dissections non-operatively. Our current outpatient management algorithm for patients with supra-aortic dissection without evidence of rupture or end-organ malperfusion includes anti-impulse beta-blocker and calcium channel blocker therapy with target systolic pressure of less than 120 mmHg and heart rate of less than 80 beats per minute, an antiplatelet regimen with aspirin, and close follow-up with CTA imaging to monitor aneurysmal growth. The long-term management of this patient will likely include an aortic debranching with individual bypasses to the right carotid and axillary arteries once aneurysmal degeneration worsens. We believe that initial non-operative management of uncomplicated supra-aortic dissections enables surgeons to transition intervention from urgent/emergent status to a purely elective operation, potentially also improving the surgical outcomes for this rare presentation.

## Data Availability

Data sharing is not applicable to this article as no datasets were generated or analyzed during the current study.

## Abbreviations

CTA: Computed tomographic angiography
